# Crocin Alleviates Pain Hyperalgesia in AIA Rats by Inhibiting the Spinal Wnt5a/*β*-Catenin Signaling Pathway and Glial Activation

**DOI:** 10.1155/2020/4297483

**Published:** 2020-01-11

**Authors:** Jin-Feng Wang, Hai-Jun Xu, Zhao-Long He, Qin Yin, Wei Cheng

**Affiliations:** ^1^Xuzhou Central Hospital, Xuzhou 221002, China; ^2^The People's Hospital of Kizilsu Kirghiz Autonomous Prefecture, Xinjiang 845350, China; ^3^The Affiliated Hospital of Xuzhou Medical University, Jiangsu Province Key Laboratory of Anesthesiology, Jiangsu Province Key Laboratory of Anesthesiology and Center for Pain Research and Treatment, Xuzhou Medical University, Xuzhou 221002, China

## Abstract

At present, most of the drugs have little effect on the pathological process of rheumatoid arthritis (RA). Analgesia is an important measure in the treatment of RA and is also one of the criteria to determine the therapeutic effects of the disease. Some studies have found that crocin, a kind of Chinese medicine, can effectively alleviate pain sensitization in pain model rats, but the mechanism is not clear. Emerging evidence indicates that crocin may inhibit the metastasis of lung and liver cancer cells from the breast by inhibiting Wnt/*β*-catenin and the Wnt signaling pathway is closely related to RA. Wnt5a belongs to the Wnt protein family and was previously thought to be involved only in nonclassical Wnt signaling pathways. Recent studies have shown that Wnt5a has both stimulatory and inhibitory effects on the classical Wnt signaling pathway, and so, Wnt5a has attracted increasing attention. This study demonstrated that crocin significantly increased the mechanical thresholds of adjuvant-induced arthritis (AIA) rats, suggesting that crocin can alleviate neuropathic pain. Crocin significantly decreased the levels of pain-related factors and glial activation. Foxy5, activator of Wnt5a, inhibited the above effects of crocin in AIA rats. In addition, intrathecal injection of a Wnt5a inhibitor significantly decreased hyperalgesia in AIA rats. This research shows that crocin may alleviate neuropathic pain in AIA rats by inhibiting the expression of pain-related molecules through the Wnt5a/*β*-catenin pathway, elucidating the mechanism by which crocin relieves neuropathic pain and provides a new way of thinking for the treatment of AIA pain.

## 1. Introduction

Rheumatoid arthritis (RA) is characterized by joint pain and deformity caused by chronic degenerative changes in articular cartilage, which seriously affect the quality of life. At present, most drugs for rheumatoid arthritis have little effect on the pathological process of RA. Analgesia is an important measure in the treatment of RA and is also one of the criteria to determine the therapeutic effects of the disease [1, 2].

The Wnt signaling pathway is a complex and ancient signal transduction system within and between cells. It is closely related to many important physiological functions, such as rheumatoid arthritis, stress, injury, repair, immunity, remodeling, and metabolism [3].

Although the Wnt signaling pathway has only recently been studied in RA, it can act on the pathological process of RA in various ways and play an important role in the occurrence and development of RA. The Wnt/*β*-catenin signaling pathway is a classical Wnt signaling pathway, and it is also the most detailed and important Wnt signaling pathway currently studied. The Wnt/*β*-catenin pathway can affect the process and prognosis of RA by affecting the proliferation of fibroblast-like synoviocytes and regulating the secretion of inflammatory factors and bone metabolism/bone destruction [4–7].

Wnt proteins can activate classical (*β*-catenin dependent) or nonclassical (*β*-catenin independent) Wnt signaling pathways, both of which play important roles in physiological and pathological processes. Wnt5a belongs to the Wnt protein family and was previously thought to be involved only in nonclassical Wnt signaling pathways. Recent studies have shown that Wnt5a has both stimulatory and inhibitory effects on the classical Wnt signaling pathway, and so, Wnt5a has attracted increasing attention [8–10].

During the development of the nervous system, the Wnt protein family plays an important role in regulating axon regeneration and in the pathogenesis of neuropathic pain. Wnt5a plays an important role in central sensitization and neuronal plasticity in acute and chronic pain [11–13].

The expression of Wnt5a and its receptor in the spinal cord is upregulated in spinal nerve ligation (SNL) mice [14], HIV-gp120 [15, 16] or capsaicin-induced pain models [17], and experimental autoimmune encephalomyelitis (EAE) mice. The specific Wnt5a inhibitor Box5 can attenuate the mechanical hyperalgesia induced by CCI [18, 19]; conversely, the Wnt5a activator Foxy5 can promote mechanical hyperalgesia [20].

Activation of the Wnt signaling pathway can stimulate production of the proinflammatory factors TNF-*α* and IL-1*β* and exacerbate the progress of neuropathic pain [21–23]. In addition, COX-2, a downstream target molecule of the Wnt5a/*β*-catenin pathway [24, 25], can affect cell function by regulating the expression of iNOS.

It is known that neuropathic pain and RA pain have similar molecular mechanisms. Emerging evidence suggests that Wnt5a in the spinal cord may play an important role in the regulation of chronic pain and RA. However, it is unclear whether the Wnt5a pathway in the spinal cord plays a role in rheumatoid arthritis pain.

In recent years, Chinese medicine has made some progress in anti-AIA pain and has become one of the hotspots of this research. Crocin is a chemical constituent extracted from saffron (a traditional Chinese medicine). *Crocus sativus* not only lowers blood pressure, regulates lipids and immunity, and protects the liver and kidney but also has analgesic effects. Its main analgesic component is crocin [26–29].

Crocin is a kind of polyhydroxy flavonoid with anti-inflammatory, antioxidant, and antidepressant effects [26]. In recent years, some studies have found that crocin can effectively alleviate pain sensitization in CCI and STZ model rats [30–34], but the mechanism is not yet clear.

In previous studies of triple-negative breast cancer (TNBC), crocin inhibited the metastasis of lung and liver cancer cells from the breast by inhibiting Wnt/*β*-catenin and increased the weight, survival rate, and volume of tumors in mice. Crocin reduced metastasis, invasion, and extracellular adhesion of TNBC cells in a dose-dependent manner, and its mechanism was related to the inhibition of the downstream factors FZD7, NEDD9, VIM, and VEGF-a [35, 36].

Combined with the above studies, we hypothesize that crocin plays role against rheumatoid pain by regulating the Wnt5a/*β*-catenin pathway. In this study, we established an AIA rat model to investigate the effect of crocin on AIA pain and its relationship with Wnt5a/*β*-catenin and its downstream factors.

## 2. Materials and Methods

### 2.1. Materials

SPF male SD rats (8-10 weeks old, weighing 180-220 g) were provided by the Laboratory Animal Center of Xuzhou Medical University. All animal experiments were approved by the Animal Ethics Committee of Xuzhou Medical University. Crocin (Sigma); Von Frey (Institute of Bioengineering, Chinese Academy of Medical Sciences); mouse anti-Wnt5a, *β*-catenin/COX-2 and iNOS polyclonal antibodies, and sheep anti-mouse antibody (HRP) were obtained from Abcam (UK); ELISA kits (TNF-*α*, IL-1*β* (eBioscience, Vienna, Austria)) were used.

### 2.2. Experimental Method

#### 2.2.1. Animal Model and Intrathecal Catheter [37]

After anesthesia, the interval between the lumbar 4 and lumbar 3 spinous processes was exposed. Two centimeters of a clear cerebral PE-10 catheter (15 cm in length, 10 *μ*l in volume) was inserted through the rupture. An 18G epidural puncture needle was passed through a subcutaneous tunnel in the rats. The other end of the catheter was sent to the back of the neck. Two centimeters of the catheter was exposed and fixed. The external opening was sealed with a lighter to prevent cerebrospinal fluid spillover. Penicillin was injected intramuscularly 3 days after the operation to prevent infection. The rats with spinal cord injury were not included in the experiment. On the second day after the operation, 2% lidocaine hydrochloride (20 *μ*l) was injected through the PE-10 catheter. Paralysis of both hind feet of the rats was soft and recovered in approximately 30 minutes; thus, the intrathecal catheterization was successful.

### 2.3. AIA Model Establishment [38]

After 7 days of intrathecal catheterization, the rats were anesthetized and intradermally injected with 0.1 ml Freund's complete adjuvant (CFA) (1 mg/ml of heat-inactivated Mycobacterium tuberculosis dissolved in 85% paraffin oil and 15% mannide monooleate; Sigma) to establish the adjuvant arthritis animal. The control group was injected with 0.1 ml saline.

The mechanical withdrawal threshold (MWT) was measured 1 day before and after the operation. The AIA model was considered successful when MWT decreased by more than 40% on the 8th day after the operation compared with the baseline pain threshold.

### 2.4. Drug Treatment

According to the preexperiment and previous study [33], intraperitoneal injections of crocin at doses of 50 and 100 mg/kg were selected in our study, while the other medicines were intrathecally injected into the different groups. The rats in each group were decapitated and killed after the tests. The ip injections of crocin were performed 30 min before injections of other drugs. The spinal cord tissues of L4-L6 were immediately acquired for subsequent experiments.

### 2.5. The Degree of Toe Swelling

One day before CFA injection and at different time points after injection, the toe swelling of the rat was detected with a toe volume measuring instrument. The difference between the volume after inflammation at a certain time and the volume before CFA injection was the swelling of the foot at that time degree.

### 2.6. Von Frey Test

The rats were placed in a transparent box with wire mesh at the bottom. They were allowed to acclimate for 30 minutes before the experiment. Von Frey filaments (0.6, 1, 2, 4, 6, 8, 10, and 15 g) were used to test the pain threshold by the up-down method. The intensity increased slowly and uniformly. If the rats had contractions or foot licking, a positive reaction was observed. At this time, the MWTs were recorded.

### 2.7. ELISA

The spinal cord tissue of each group was homogenized with a microgrinder. The homogenate was diluted 30 times, and the levels of TNF-*α* and IL-1*β* were detected according to ELISA kit instructions.

### 2.8. Western Blotting to Detect Protein Expression

After boiling for 5 minutes, 80 *μ*g of protein was separated by SDS-PAGE. Then, the proteins were transferred to a PVDF membrane at 150 mA for 3 hours. The membrane was blocked in 3% skimmed milk powder for 1 hour, incubated with primary antibody (all 1 : 1 000) overnight at 4°C, and washed with TBST (5 min × 3 times). After incubation with the secondary antibody at room temperature for 1 hour, TBST was used to wash the membrane (5 min × 3 times), and ECL was added for exposure and development.

### 2.9. Immunofluorescence Staining

After anesthesia, the rat hearts were exposed rapidly. After ascending aorta intubation, the blood was washed with 200 ml normal saline and then perfused with 4% polyformaldehyde phosphate buffer (0.1 MB, pH 7.4) (200 ml). The L_4-5_ segments were fixed in 4% paraformaldehyde for 4-6 hours (4°C) and then immersed in 30% sucrose solution at 4°C overnight until they sank. Frozen coronal serial sections were collected in 0.01 PBS. The sections were washed at room temperature 3 times for 5 min, incubated at room temperature with fluorescent sealing fluid for 2 hours, dried, and incubated overnight with polyclonal goat anti-rat primary antibody (Abcam, USA) at 4°C. The sections were washed with PBS 3 times for 5 min, and fluorescent-labeled donkey anti-goat FITC (1 : 100, Biyun Tian) secondary antibody was added in the darkroom and incubated at room temperature in the dark. After 2 hours, the sections were washed with PBS in the darkroom 3 times for 5 min, covered, and fluorescently sealed, and confocal microscopy (TCS-SP2, Leica, Germany) was used to observe and take pictures.

### 2.10. Statistical Analysis

Data are expressed as the mean ± SEM. SPSS 16.0 statistical software was used to test the difference between groups. ANOVA (Tukey, SNK), two-way ANOVA, and repeated measures ANOVA were employed to test the data, and *P* < 0.05 indicated that the difference was significant.

## 3. Results

### 3.1. The Establishment of AIA Model

**Figure 1:**
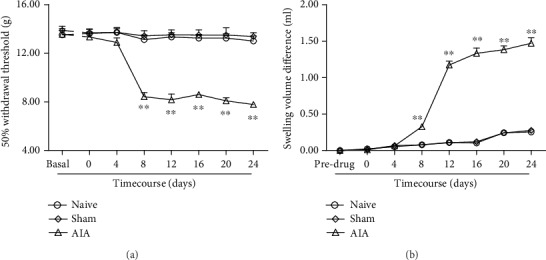
(a) After model establishment, mechanical allodynia was observed in the AIA model. Sham rats did not show a decrease in MWTs. ^∗∗^*P* < 0.01*vs*. day 0 (mean ± SEM, *n* = 8). (b) The paw swelling of AA rats. ^∗∗^*P* < 0.01*vs*. predrug value in AA rats (mean ± SEM, *n* = 8). Swelling volume difference = Volume (pre‐CFA injection) − Volume (post‐CFA injection) (mean ± SEM, *n* = 8).

There was no significant difference in MWTs between the normal and sham groups (*P* > 0.05). The MWTs were significantly decreased in AIA rats (*P* < 0.05, Figure 1(a)). The results showed that the operation induced mechanical hyperalgesia in rats. Along with the course extension, paw swelling of AA rats increased gradually. Paw swelling is still obvious on day 24 (*P* < 0.05, Figure 1(b)).

### 3.2. Intraperitoneal Injection of Crocin Can Significantly Alleviate AIA-Induced Mechanical Pain

**Figure 2:**
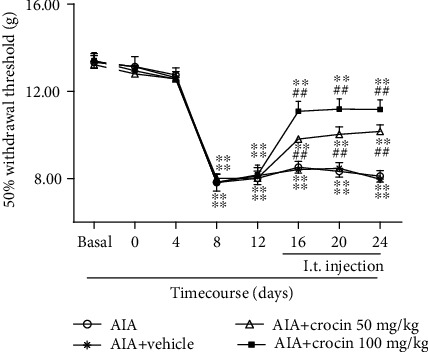
Changes in MWTs in AIA rats after injection of crocin (mean ± SEM, *n* = 8). ^∗∗^*P* < 0.01*vs*. basal; ^##^*P* < 0.01 (AIA+crocin groups *vs*. AIA+vehicle).

Crocin had no significant effects in the sham group (*P* > 0.05). Crocin significantly increased the MWTs in AIA rats (*P* < 0.05, Figure 2). The results showed that crocin may induce analgesic effects in AIA rats.

### 3.3. Crocin Reduces the Expression of Inflammatory Factors and Pain-Related Molecules in the Spinal Dorsal Horn of AIA Rats

**Figure 3:**
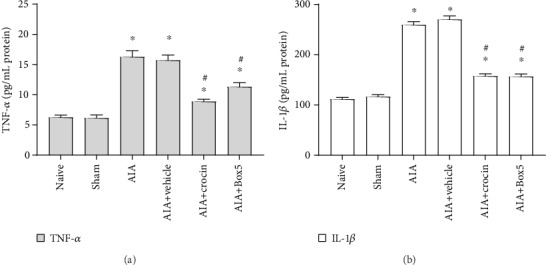
Changes of spinal TNF-*α* and IL-1*β* after injection of crocin in AIA rats (mean ± SEM, *n* = 8). ^∗^*P* < 0.05, compared with the sham group; ^#^*P* < 0.05, compared with the AIA+vehicle group (*vs*. AIA+crocin group or AIA+Box5 group).

ELISA results showed that crocin significantly decreased the levels of TNF-*α* (*P* < 0.05, Figure 3(a)) and IL-1*β* (*P* < 0.05, Figure 3(b)) in the spinal cords of AIA rats.

### 3.4. Crocin Inhibits the Wnt5a/*β*-Catenin Pathway and Glial Activation

**Figure 4:**
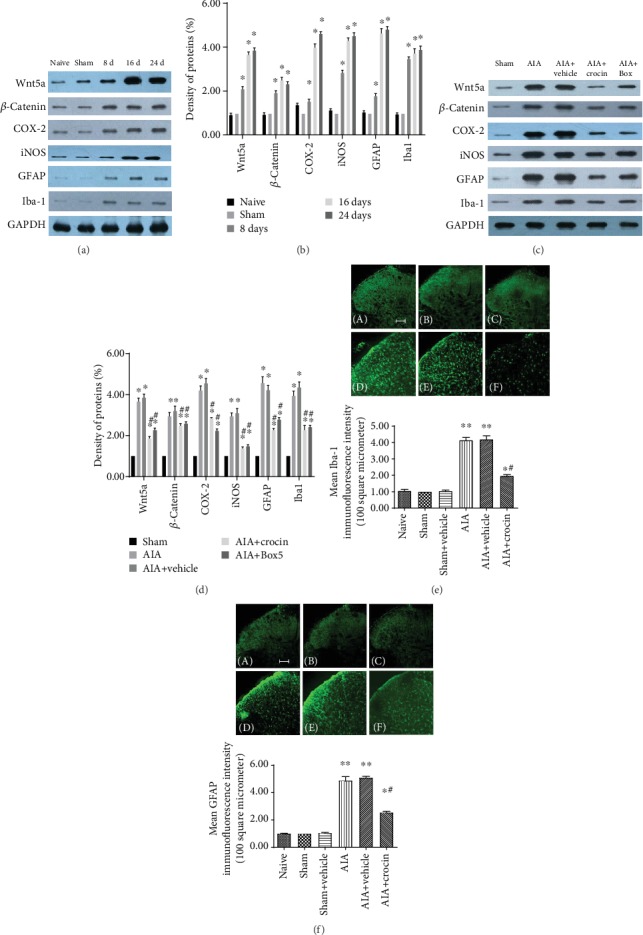
The effect of crocin on expression of the Wnt5a/*β*-catenin pathway and glial cell activation in AIA rats. (a–d) Western blot results (mean ± SEM, *n* = 3); ^∗^*P* < 0.05, compared with the sham group; ^#^*P* < 0.05, compared with the AIA+vehicle group (*vs*. AIA+crocin group). (e, f) AIA model and crocin effects on glial cell activation (mean ± SEM, *n* = 6); ^∗^*P* < 0.05, compared with the sham+vehicle group; ^#^*P* < 0.05, compared with the AIA+vehicle group (*vs*. AIA+crocin group).

Western blot results showed that upregulation of the Wnt5a/*β*-catenin pathway and glial cell marker proteins was observed in the spinal cords of AIA rats (Figures 4(a)–4(d)). The use of crocin may decrease expression of the Wnt5a/*β*-catenin pathway and glial activation in the spinal cords of AIA rats.

The levels of Iba-1 and GFAP in the dorsal horn of the spinal cord were significantly higher than those in the control group (Figures 4(e) and 4(f)). Compared with levels in the vehicle group, the increase in GFAP and Iba-1 was abrogated in the crocin group.

### 3.5. Intrathecal Injection of the Wnt5a Activator (Foxy5) on the Pain Thresholds of Rats

The above data proved that crocin attenuated pain facilitation and the Wnt5a pathway in the spinal dorsal horn of AIA rats. We further observed the intrathecal injection of the Wnt5a activator (Foxy5) on crocin-induced analgesia in AIA rats.

**Figure 5:**
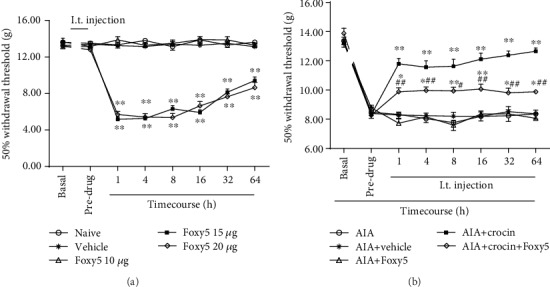
Intrathecal injection of the Wnt5a activator (Foxy5) on the MWTs in rats (mean ± SEM, *n* = 8). ^∗^*P* < 0.05, ^∗∗^*P* < 0.01, *vs*. predrug; ^#^*P* < 0.05, ^##^*P* < 0.01 (AIA+crocin groups *vs*. AIA+Foxy5).

Vehicle and Foxy5 (10 *μ*g) did not affect the basal MWTs in rats, while Foxy5 (15, 20 *μ*g) may lead to pain facilitation in normal rats. Compared with the effect in the AIA+crocin group, the analgesic effect of crocin (100 mg/kg) was attenuated in the Foxy5 (10 *μ*g)+AIA+crocin group (Figure 5).

### 3.6. Intrathecal Injection of the Wnt5a Inhibitor Can Alleviate Mechanical Hyperalgesia

The above data proved that the Wnt5a activator alleviated crocin-induced analgesia in AIA rats. Then, we observed the effects of the Wnt5a inhibitor (Box5) on the pain thresholds of AIA rats.

**Figure 6:**
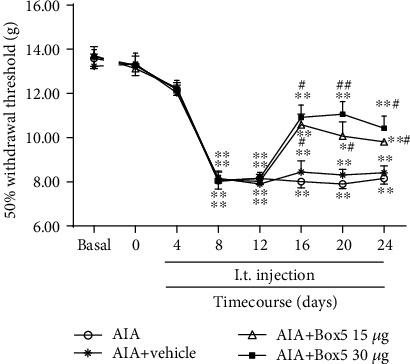
Effects of the Wnt5a inhibitor (Box5) on the MWTs in AIA rats (mean ± SEM, *n* = 8). ^∗^*P* < 0.05, ^∗∗^*P* < 0.01*vs*. basal value; ^#^*P* < 0.05, ^##^*P* < 0.01 (AIA+Box5 groups *vs*. AIA+vehicle).

Intrathecal injection of Box5 blocked pain hypersensitivity in AIA rats compared with the vehicle group (Figure 6).

### 3.7. Distribution of Wnt5a/*β*-Catenin in the Spinal Dorsal Horn of AIA Rats

**Figure 7:**
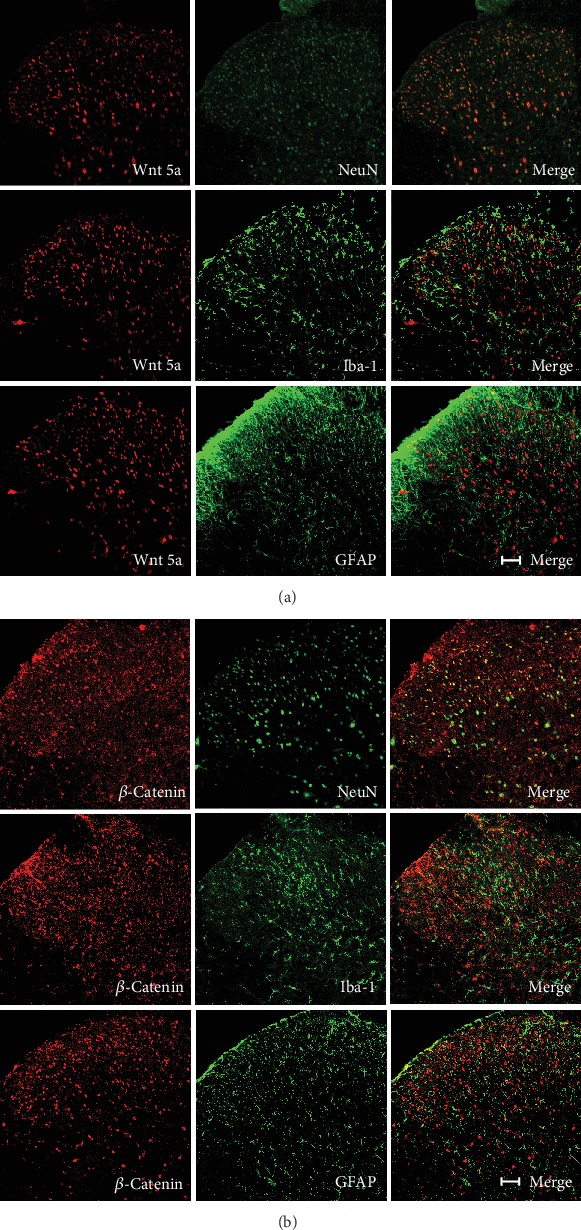
Distribution of the Wnt5a/*β*-catenin pathway in the spinal dorsal horn of AIA rats (scale bar = 100 *μ*m).

Neurons, astrocytes, microglial markers (NeuN, GFAP, and Iba-1), and Wnt5a/*β*-catenin pathway proteins were used for fluorescence double labeling. The expression of Wnt5a/*β*-catenin was not detected in nearly any of the GFAP and Iba-1 IR cells. However, positive expression of Wnt5a/*β*-catenin was detected in NeuN IR cells. This suggests that Wnt5a/*β*-catenin is mainly distributed in the dorsal horn of AIA rats (Figures 7(a) and 7(b)).

## 4. Discussion

RA is a chronic, systemic autoimmune disease characterized by symmetry and multijoint inflammation that seriously affects the quality of life [38]. Arthritis-related joint pain leads to limited movement, weakness, severe pain, and even serious impacts on daily life. The application of oral analgesics is limited by inadequate pain relief, severe adverse reactions, and other drug interactions; intra-articular injection of corticosteroids and viscoelastics can relieve pain, but the effects and duration are unstable; the prognosis of arthroscopic debridement is not better than that of placebo or chemical therapy; and joint replacement has a high surgical risk. Therefore, there is an urgent need for a safe and effective method for the treatment of chronic arthritis in AIA. In this study, we found that the mechanical pain threshold of AIA model rats decreased significantly after injection of CFA, which means that the modeling was successful.

Crocin, as a typical flavonoid, has antiviral, hypoglycemic, hypotensive, immunomodulatory, and cardiovascular protective activities [39–41]. In recent years, studies have shown that crocin has protective effects against alcohol-induced neurological damage in mice. Crocin has been found to increase the pain threshold in models [30–34], but its mechanism is not well studied.

This study demonstrated that crocin significantly increased the mechanical thresholds of AIA rats, suggesting that crocin can alleviate neuropathic pain in AIA rats. Crocin significantly decreased the levels of pain-related factors (Wnt5a and *β*-catenin, TNF-*α* and IL-1*β*) and glial activation. Foxy5 (an activator of Wnt5a) inhibited the above effects of crocin in AIA rats. In addition, intrathecal injection of a Wnt5a inhibitor significantly decreased hyperalgesia in AIA rats.

It is known that expression levels of the inflammatory factors TNF-*α* and IL-1*β* are significantly increased in the chronic sciatic nerve constriction injury model [42, 43], which is consistent with the present results. A high concentration of TNF-*α* in the central nervous system can be regarded as neurotoxic and can induce the production of oxygen free radicals in the central nervous system. Previous studies also proved that inhibition of inflammatory signaling pathways can effectively alleviate neuropathic pain [44–49].

Wnt5a has been reported to play an important role in the inflammatory response. It can upregulate the expression of many important proinflammatory factors and inflammatory mediators, including interleukin-1*β* (IL-1*β*), interleukin-6 (IL-6), and tumor necrosis factor alpha (TNF-*α*) [44]. Zhang et al. proved that the expression of inflammatory cytokines in the spinal cord increased after CCI surgery, and activation of the Wnt/*β*-catenin pathway induced by Wnt agonists could also increase the expression of inflammatory cytokines in the spinal cord [42]. Li et al. observed that Wnt5a played an important role in the regulation of TNF-*α* and IL-1*β* expression levels by culturing mixed neurons [43]. The previously mentioned studies were consistent with the present results. Therefore, we proved for the first time that crocin alleviates AIA pain in rats by inhibiting Wnt5a/*β*-catenin and the downstream inflammatory pathway, but the specific mechanism requires further experimental study.

The Wnt signaling pathway is involved in the regulation of chronic pain, which may be related to spinal dorsal horn neuroinflammation. Marchetti and Pluchino showed that the Wnt signaling pathway participates in the production of the inflammatory response in the central nervous system mediated by glial cell modification [50]. Halleskog et al. showed that many Wnt receptor proteins are expressed in microglia (such as N13 cells and primary mouse microglia). Another experiment showed that exogenous Wnt proteins activate classical or nonclassical Wnt signaling pathways in microglia. Inhibitors of the Wnt/*β*-catenin signaling pathway administered by intrathecal injection significantly reduced PSL-induced abnormal pain and abnormal activation of glial cells in the spinal dorsal horn [51], suggesting that the Wnt signaling pathway is closely related to abnormal activation of glial cells in neuropathic pain. Activation of microglia induced by neuroinflammation and subsequent release of a series of proinflammatory cytokines plays an important role in inducing hyperalgesia through central sensitization [52–54].

Moreover, COX-2, a target molecule downstream of the Wnt/*β*-catenin pathway, can affect cell function by regulating the expression of iNOS. NO is a new neurotransmitter and inflammatory mediator that is catalyzed by nitric oxide synthase. It can be categorized as endothelial nitric oxide synthase (eNOS/NOS3), neuronal NOS (nNOS/NOS1), and inducible NOS (iNOS/NOS2). NO can also enhance the sensitivity of pain perception and transmission. Excessive concentrations of NO can lead to increased pain and inflammatory responses in the spinal cord center [55–57]. Animal experiments showed that COX-2, as a pain-related gene, was overexpressed in a neuropathic pain model. Therefore, selective COX-2 inhibitors can effectively inhibit the development of neuropathic pain [58–62].

Our results showed that crocin significantly decreased the expression levels of iNOS and COX-2 in the spinal cords of AIA rats. These results suggest that crocin may reduce the expression of the inflammatory factors and pain-related molecules iNOS and COX-2 in the spinal cord of AIA rats by inhibiting the Wnt/*β*-catenin pathway.

In conclusion, the results showed that crocin may alleviate neuropathic pain in AIA rats by inhibiting the expression of pain-related molecules through the Wnt5a/*β*-catenin pathway. This study elucidates the mechanism by which crocin alleviates neuropathic pain and provides a new way of thinking for the treatment of AIA pain.

## Data Availability

The data used to support the findings of the present research are available from the corresponding authors once requested.
